# Redetermination of the crystal structure of 2-oxo-1,3-thia­zolidin-4-iminium chloride

**DOI:** 10.1107/S2056989019003189

**Published:** 2019-03-11

**Authors:** Manickam Muthukkumar, Ammasai Karthikeyan, Madeshwaran Poovarasan, Vadivel Ruckmani, Dhanakotti Rajaram, Samson Jegan Jennifer, Ibrahim Abdul Razak

**Affiliations:** a Department of Chemistry, Selvamm Arts and Science College, Namakkal, Tamilnadu, India; b Department of Chemistry, St. Joseph University, Nagaland 797 115, India; c X-ray Crystallography Unit, School of Physics, Universiti Sains Malaysia, 11800, USM, Penang, Malaysia

**Keywords:** crystal structure, thio­urea, chloro­acetic acid, thia­zolidine, hydrogen bonding

## Abstract

In the redetermination of the title compound, C_3_H_5_N_2_OS^+^·CI^−^, the asymmetric unit consists of one independent 2-oxo-1,3-thia­zolidin-4-iminium cation and one independent chloride anion. The cation inter­acts with a chloride anion *via* N—H⋯Cl hydrogen bonds forming a supra­molecular chain along [010]. These supra­molecular chains are further extended by weak C—H⋯Cl and C—H⋯O inter­actions, forming a two-dimensional network parallel to (001). The crystal structure is further stabilized by weak C—O⋯π inter­actions, supporting a three-dimensional architecture. The structure was previously determined by Ananthamurthy & Murthy [*Z. Kristallogr.* (1975). **8**, 356–367] but has been redetermined with higher precision to allow the hydrogen-bonding patterns and supra­molecular inter­actions to be investigated.

## Abstract 2

2-Oxo-1,3-thia­zolidin-4-iminium cations inter­act with a chloride anion *via* N—H⋯Cl hydrogen bonds, forming a supra­molecular chain. These supra­molecular chains are further extended by weak C—H⋯Cl, C—H⋯O and C—O⋯π inter­molecular inter­actions forming a 3D supra­molecular network.

## Chemical context   

Thio­urea and its derivatives are an important group of organic compounds because of their diverse application in fields such as medicine, agriculture, coordination, and analytical chemistry (Saeed *et al.*, 2010 ▸, 2014 ▸). The complexes with thio­urea derivatives expressing biological activity have been successfully screened for various biological actions such as anti­bacterial, anti­fungal, anti­cancer, anti­oxidant, anti-inflam­matory, anti­malarial, anti­viral activity, as anti-HIV agents and also as catalysts (Saeed *et al.*, 2010 ▸). Thia­zolidine derivatives show anti­tumor activity as well as a broad range of biological activities including anti­bactericidal, fungicidal, anti-angiogenesis, anti­diabetic and anti­microbial (Singh *et al.*, 1981 ▸; Saeed & Florke, 2006 ▸; Rizos *et al.*, 2016 ▸). Thio­urea derivatives are used as phase-change materials for thermal energy storage (Alkan *et al.*, 2011 ▸). In addition, metal complexes of thio­urea derivatives are also studied for their relationship to NLO materials (Rajasekaran *et al.*, 2003 ▸; Ushasree *et al.*, 2000 ▸). Thio­urea derivatives find applications related to their uses as synthons in supra­molecular chemistry (Saeed & Florke, 2006 ▸). Organic and inorganic complexes of thio­urea derivatives form well-defined non-covalent supra­molecular architectures *via* multiple hydrogen bonds involving the N, S and O atoms. We report herein the mol­ecular structure and supra­molecular architecture of the title salt, C_3_H_5_N_2_SO^+^CI^−^, (I), formed from the reaction of thio­urea with mono­chloro acetic acid. A determination of this crystal structure was performed by Ananthamurthy & Murthy (1975 ▸). However, while the authors could identify the space group as *Pbca* and determine the cell parameters [*a* = 9.53 (1), *b* = 17.61 (5), *c* = 7.71 (1) Å], these were not accurate enough to examine the hydrogen-bonding patterns and supra­molecular inter­actions that are described here.
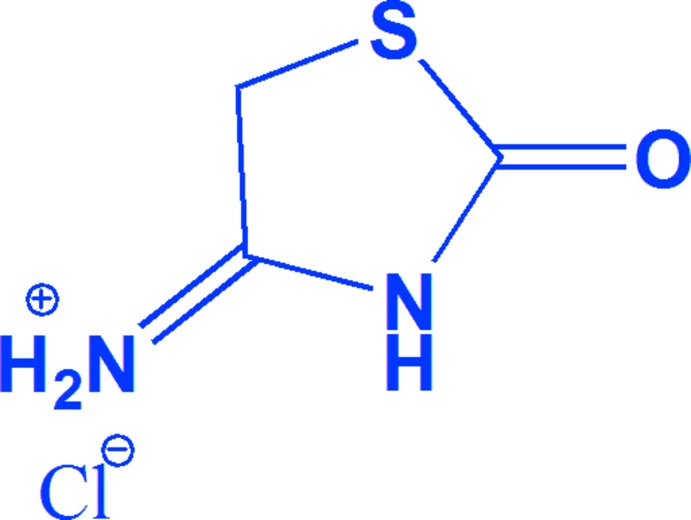


## Structural commentary   

**Figure 1:**
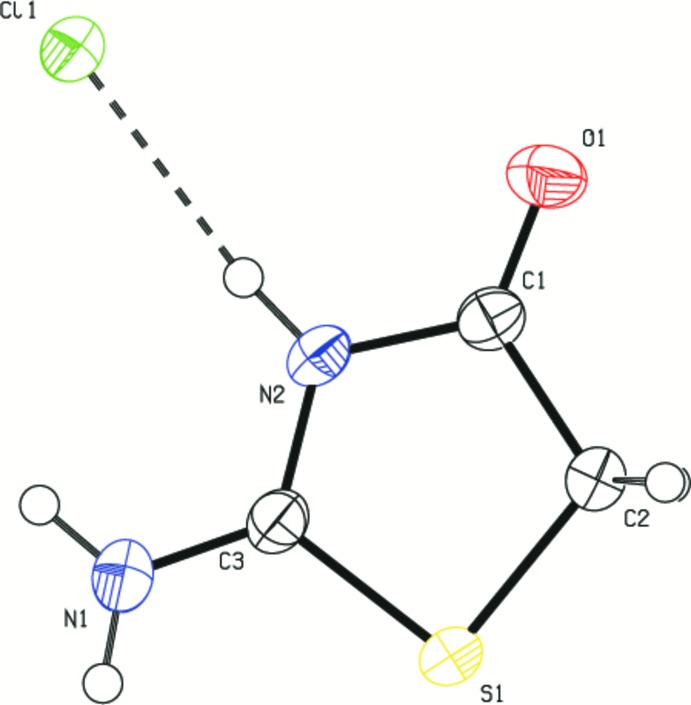
Asymmetric unit of the title compound, showing the atom-numbering scheme and 50% probability displacements ellipsoids. The dashed line represents the N2—H4⋯Cl1 hydrogen bond.

The asymmetric unit of the title compound (I) consists of one 2-imino-4-oxo-1,3-thia­zolidine cation and one hydro­chloride anion (Fig. 1 ▸). In the cation, the C3=N1 bond has double-bond character. The C3—N1 and C3—N2 bond distances indicate tautomerism between the amino N1 and imino N2 groups. The exocylic bond [C3—N1 = 1.2930 (17) Å] is short and its length is comparable with that of the endocylic C3—N2 bond [1.3432 (16) Å], confirming the C3=N1 double-bond assignment. The bond lengths and angles agree with those reported for similar structures (Ananthamurthy & Murthy, 1975 ▸; Xuan *et al.*, 2003 ▸; Vedavathi & Vijayan, 1981 ▸).

## Supra­molecular features   

**Figure 2:**
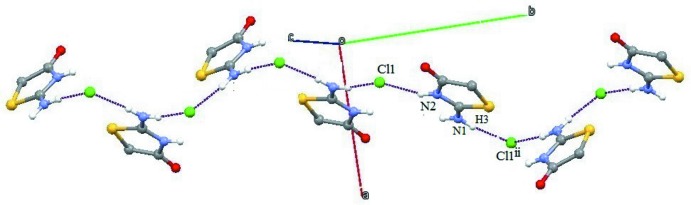
A view of a chain formed by N—H⋯Cl hydrogen bonds (dashed lines). Symmetry code as in Table 1 ▸.

**Figure 3:**
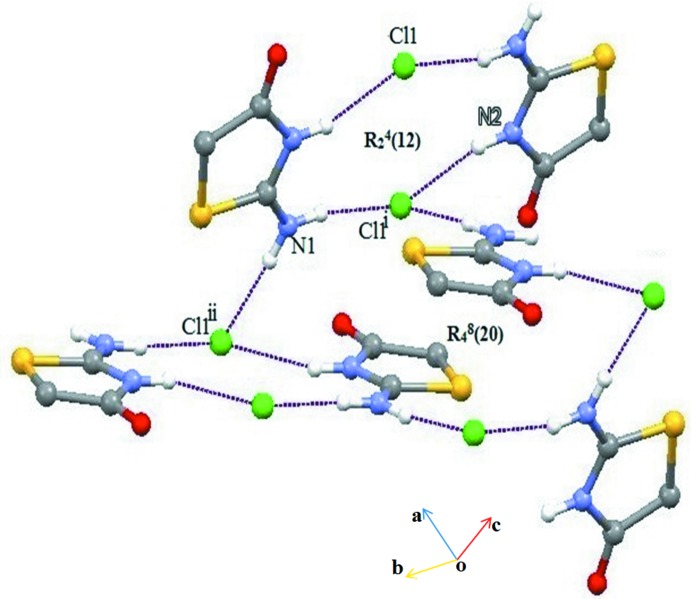
A view of the two supra­molecular 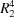
(12) and 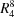
(20) ring motifs in the structure of (I), formed by N—H⋯Cl hydrogen bonds (dashed lines). Symmetry codes are given in Table 1 ▸.

**Figure 4:**
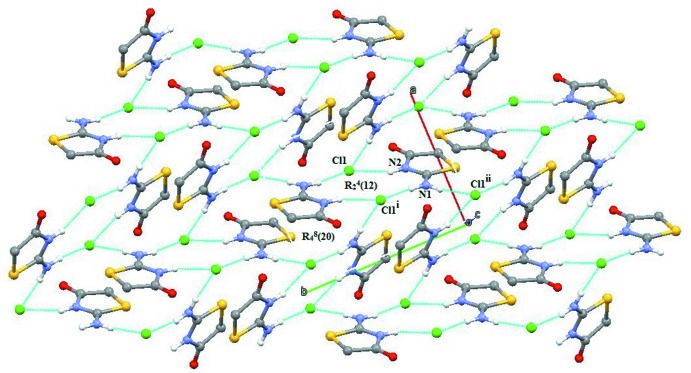
A view of the supra­molecular sheet-like structures within the crystal packing of (I). Green dashed lines indicate N—H⋯Cl hydrogen bonds. Symmetry codes are given in Table 1 ▸.

**Figure 5:**
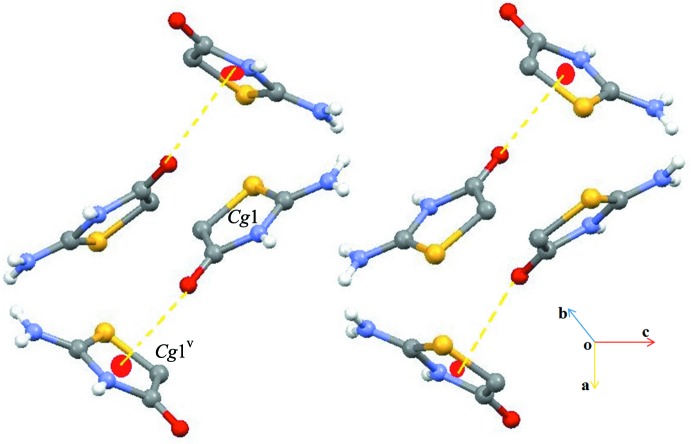
A view of the weak C—O⋯π inter­actions (dashed lines) in (I). *Cg*1 is the centroid of the thia­zolidine ring. Symmetry codes are given in Table 1 ▸.

**Figure 6:**
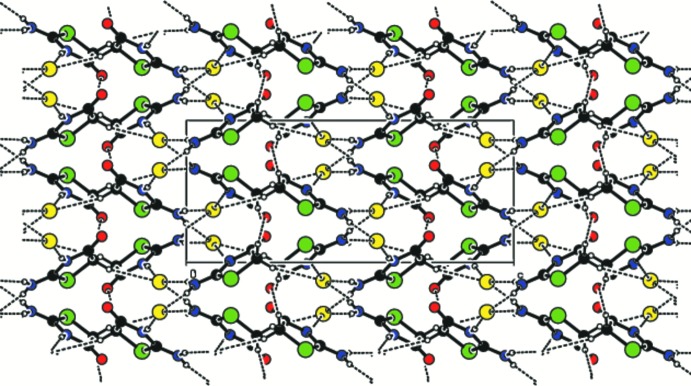
A view of the three-dimensional architecture of the title compound.

The 2-imino-4-oxo-1,3-thia­zolidine cation inter­acts with the chlorine anion in the asymmetric unit *via* the N2—H4⋯Cl hydrogen bond (Table 1 ▸) and with symmetry-related Cl^−^anions *via* N1—H1⋯Cl and N1—H3⋯Cl hydrogen bonds, forming supra­molecular chains along [010] (Fig. 2 ▸). The chlorine anion inter­acts with the N2 atom and the exocyclic N1 atom of the thia­zolidine moiety through the N2—H4⋯Cl hydrogen bond and the pair of N1—H1⋯Cl and N1—H3⋯Cl hydrogen bonds, forming 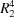
(12) ring motifs in the [010] plane (Fig. 3 ▸). This motif is further connected on the other side by 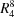
(20) ring motifs, generating a sheet-like structure parallel to (001) (Fig. 4 ▸). The supra­molecular sheets and crystal packing are further stabilized by weak C—H⋯Cl, C—H⋯O and C=O⋯π inter­actions (Table 1 ▸, Fig. 5 ▸). All of these inter­actions combine to generate a three-dimensional supra­molecular architecture (Fig. 6 ▸).

## Database survey   

The crystal structures of a number of related and substituted thio­urea derivatives and thia­zoline salts and their metal complexes have also been investigated in a variety of crystalline environments. These include dl-2-amino-2-thia­zoline-4-carb­oxy­lic acid trihydrate (Xuan *et al.*, 2003 ▸), 2-amino-1,3-thia­zoline hydro­chloride (Vedavathi & Vijayan, 1981 ▸), *N*-(4-chloro­benzo­yl)-*N*,*N*-di­phenyl­thio­urea (Arslan *et al.*, 2003*a* ▸), 1-(4-chloro-benzo­yl)-3-naphthalen-1-yl-thio­urea (Arslan *et al.*, 2003*b* ▸) and 1–(4–chloro­phen­yl)–3–(4–μethyl­benzo­yl)thio­urea (Saeed & Floörke, 2006 ▸). N—H⋯Cl hydrogen bonds play a major role in building up the supra­molecular architectures of many related crystal structures (for examples, see: Diallo *et al.*, 2014 ▸; Yamuna *et al.*, 2014 ▸; Plater & Harrison, 2016 ▸; Khongsuk *et al.*, 2015 ▸).

## Synthesis and crystallization   

Hot ethanol solutions of thio­urea (32 mg) and chloro acetic acid (37 mg) were mixed in a 1:1 molar ratio. The resulting solution was warmed over a water bath for half an hour and then kept at room temperature for crystallization. After a week, light-yellow prismatic crystals suitable for single-crystal X-ray analysis were obtained.

## Refinement   

Crystal data, data collection and structure refinement details are summarized in Table 2 ▸. All H atoms were initially located in difference-Fourier maps and were subsequently treated as riding atoms in geometrically idealized positions, with C—H = 0.93 and N—H = 0.86 and with *U*_iso_(H) = 1.2*U*_eq_(C,N).

## Supplementary Material

Crystal structure: contains datablock(s) I. DOI: 10.1107/S2056989019003189/jj2207sup1.cif

Structure factors: contains datablock(s) I. DOI: 10.1107/S2056989019003189/jj2207Isup2.hkl

Supporting information file. DOI: 10.1107/S2056989019003189/jj2207Isup3.cml

CCDC reference: 1901297

Additional supporting information:  crystallographic information; 3D view; checkCIF report

## Figures and Tables

**Table 1:** Hydrogen-bond geometry (Å, °) *Cg*1 is the centroid of the S1/N1/C1–C3 ring.

*D*—H⋯*A*	*D*—H	H⋯*A*	*D*⋯*A*	*D*—H⋯*A*
N1—H1⋯Cl1^i^	0.86	2.32	3.1484 (15)	162
N1—H3⋯Cl1^ii^	0.86	2.34	3.1903 (15)	170
N2—H4⋯Cl1	0.86	2.26	3.1026 (12)	166
C2—H2⋯Cl1^iii^	0.97	2.78	3.7137 (14)	163
C2—H5⋯O1^iv^	0.97	2.57	3.5190 (18)	165
C1—O1⋯*Cg*1^v^	1.20 (1)	3.13 (1)	3.9430 (15)	125 (1)

Symmetry codes: (i) 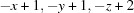; (ii) 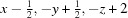; (iii) 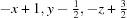; (iv) 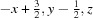; (v) 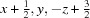.

**Table 2:** Experimental details

Crystal data	
Chemical formula	C_3_H_5_N_2_OS^+^·Cl^−^
*M*_r_	152.60
Crystal system, space group	Orthorhombic, *P* *b* *c* *a*
Temperature (K)	296
*a*, *b*, *c* (Å)	7.5106 (11), 9.3140 (13), 17.343 (3)
*V* (Å^3^)	1213.2 (3)
*Z*	8
Radiation type	Mo *K*α
μ (mm^−1^)	0.87
Crystal size (mm)	0.54 × 0.45 × 0.25

Data collection	
Diffractometer	Bruker SMART APEXII DUO CCD area detector
Absorption correction	Multi-scan (*SADABS*; Bruker, 2009 ▸)
*T*_min_, *T*_max_	0.683, 0.832
No. of measured, independent and observed [*I* > 2σ(*I*)] reflections	7517, 1798, 1554
*R*_int_	0.020
(sin θ/λ)_max_ (Å^−1^)	0.708

Refinement	
*R*[*F* ^2^ > 2σ(*F* ^2^)], *wR*(*F* ^2^), *S*	0.027, 0.077, 1.05
No. of reflections	1798
No. of parameters	73
H-atom treatment	H-atom parameters constrained
Δρ_max_, Δρ_min_ (e Å^−3^)	0.33, −0.22

Computer programs: *APEX2* and *SAINT* (Bruker, 2009 ▸), *SHELXTL* (Sheldrick, 2008 ▸), *PLATON* (Spek, 2009 ▸), *Mercury* (Macrae *et al.*, 2008 ▸) and *PLATON* (Spek, 2009 ▸).

## supplementary crystallographic information

### Crystal data

**Table:**

C_3_H_5_N_2_OS^+^·Cl^−^	*D*_x_ = 1.671 Mg m^−^^3^
*M**_r_* = 152.60	Mo *K*α radiation, λ = 0.71073 Å
Orthorhombic, *P**b**c**a*	Cell parameters from 1800 reflections
*a* = 7.5106 (11) Å	θ = 2.4–30.2°
*b* = 9.3140 (13) Å	µ = 0.87 mm^−^^1^
*c* = 17.343 (3) Å	*T* = 296 K
*V* = 1213.2 (3) Å^3^	Prism, yellow
*Z* = 8	0.54 × 0.45 × 0.25 mm
*F*(000) = 624	

### Data collection

**Table:**

Bruker SMART APEXII DUO CCD area detector diffractometer	1798 independent reflections
Radiation source: fine-focus sealed tube	1554 reflections with *I* > 2σ(*I*)
Graphite monochromator	*R*_int_ = 0.020
phi and ω scans	θ_max_ = 30.2°, θ_min_ = 2.4°
Absorption correction: multi-scan (SADABS; Bruker, 2009)	*h* = −10→10
*T*_min_ = 0.683, *T*_max_ = 0.832	*k* = −10→13
7517 measured reflections	*l* = −24→20

### Refinement

**Table:**

Refinement on *F*^2^	Primary atom site location: structure-invariant direct methods
Least-squares matrix: full	Secondary atom site location: difference Fourier map
*R*[*F*^2^ > 2σ(*F*^2^)] = 0.027	Hydrogen site location: inferred from neighbouring sites
*wR*(*F*^2^) = 0.077	H-atom parameters constrained
*S* = 1.05	*w* = 1/[σ^2^(*F*_o_^2^) + (0.0384*P*)^2^ + 0.4007*P*] where *P* = (*F*_o_^2^ + 2*F*_c_^2^)/3
1798 reflections	(Δ/σ)_max_ = 0.001
73 parameters	Δρ_max_ = 0.33 e Å^−^^3^
0 restraints	Δρ_min_ = −0.22 e Å^−^^3^

### Special details

**Table:**

Geometry. All esds (except the esd in the dihedral angle between two l.s. planes) are estimated using the full covariance matrix. The cell esds are taken into account individually in the estimation of esds in distances, angles and torsion angles; correlations between esds in cell parameters are only used when they are defined by crystal symmetry. An approximate (isotropic) treatment of cell esds is used for estimating esds involving l.s. planes.
Refinement. Refinement of F^2^ against ALL reflections. The weighted R-factor wR and goodness of fit S are based on F^2^, conventional R-factors R are based on F, with F set to zero for negative F^2^. The threshold expression of F^2^ > 2sigma(F^2^) is used only for calculating R-factors(gt) etc. and is not relevant to the choice of reflections for refinement. R-factors based on F^2^ are statistically about twice as large as those based on F, and R- factors based on ALL data will be even larger.

### Fractional atomic coordinates and isotropic or equivalent isotropic displacement parameters (Å^2^)

**Table:**

	*x*	*y*	*z*	*U*_iso_*/*U*_eq_	
S1	0.37439 (5)	−0.01239 (4)	0.86421 (2)	0.03155 (11)	
O1	0.67766 (16)	0.25652 (11)	0.75866 (7)	0.0389 (2)	
N1	0.3380 (2)	0.18329 (15)	0.97467 (7)	0.0409 (3)	
H1	0.3567	0.2663	0.9949	0.049*	
H3	0.2747	0.1209	0.9988	0.049*	
C3	0.40497 (18)	0.15240 (14)	0.90803 (7)	0.0270 (3)	
C2	0.51618 (18)	0.03829 (13)	0.78499 (7)	0.0272 (3)	
H5	0.6172	−0.0263	0.7817	0.033*	
H2	0.4507	0.0336	0.7368	0.033*	
C1	0.57886 (18)	0.18932 (13)	0.79961 (7)	0.0264 (3)	
N2	0.50511 (14)	0.24392 (11)	0.86677 (6)	0.0268 (2)	
H4	0.5224	0.3312	0.8812	0.032*	
Cl1	0.63837 (5)	0.54422 (4)	0.91839 (2)	0.03440 (11)	

### Atomic displacement parameters (Å^2^)

**Table:**

	*U*^11^	*U*^22^	*U*^33^	*U*^12^	*U*^13^	*U*^23^
S1	0.0393 (2)	0.02303 (17)	0.03232 (19)	−0.00807 (13)	0.00667 (13)	−0.00457 (12)
O1	0.0438 (6)	0.0310 (5)	0.0418 (6)	−0.0042 (5)	0.0099 (5)	0.0066 (4)
N1	0.0593 (9)	0.0310 (6)	0.0325 (6)	−0.0057 (6)	0.0111 (6)	−0.0077 (5)
C3	0.0311 (6)	0.0224 (5)	0.0275 (6)	−0.0007 (5)	−0.0013 (5)	−0.0025 (4)
C2	0.0301 (6)	0.0255 (6)	0.0261 (6)	−0.0020 (5)	0.0015 (5)	−0.0030 (4)
C1	0.0281 (6)	0.0223 (5)	0.0289 (6)	0.0021 (5)	−0.0022 (5)	0.0028 (4)
N2	0.0312 (5)	0.0188 (5)	0.0304 (5)	−0.0014 (4)	−0.0018 (4)	−0.0022 (4)
Cl1	0.0453 (2)	0.02841 (18)	0.02950 (18)	−0.00821 (13)	0.00436 (13)	−0.00460 (12)

### Geometric parameters (Å, º)

**Table:**

S1—C3	1.7280 (13)	C3—N2	1.3432 (16)
S1—C2	1.8013 (14)	C2—C1	1.5049 (18)
O1—C1	1.2027 (17)	C2—H5	0.9700
N1—C3	1.2930 (17)	C2—H2	0.9700
N1—H1	0.8600	C1—N2	1.3865 (17)
N1—H3	0.8600	N2—H4	0.8600
			
C3—S1—C2	91.38 (6)	C1—C2—H2	110.2
C3—N1—H1	120.0	S1—C2—H2	110.2
C3—N1—H3	120.0	H5—C2—H2	108.5
H1—N1—H3	120.0	O1—C1—N2	123.48 (12)
N1—C3—N2	123.56 (12)	O1—C1—C2	125.45 (12)
N1—C3—S1	122.62 (11)	N2—C1—C2	111.06 (11)
N2—C3—S1	113.82 (9)	C3—N2—C1	116.00 (11)
C1—C2—S1	107.55 (9)	C3—N2—H4	122.0
C1—C2—H5	110.2	C1—N2—H4	122.0
S1—C2—H5	110.2		
			
C2—S1—C3—N1	−176.63 (14)	N1—C3—N2—C1	174.68 (14)
C2—S1—C3—N2	2.92 (11)	S1—C3—N2—C1	−4.87 (15)
C3—S1—C2—C1	−0.44 (10)	O1—C1—N2—C3	−176.60 (14)
S1—C2—C1—O1	179.02 (12)	C2—C1—N2—C3	4.40 (16)
S1—C2—C1—N2	−2.00 (13)		

### Hydrogen-bond geometry (Å, º)

Cg1 is the centroid of the S1/N1/C1–C3 ring.

**Table:**

*D*—H···*A*	*D*—H	H···*A*	*D*···*A*	*D*—H···*A*
N1—H1···Cl1^i^	0.86	2.32	3.1484 (15)	162
N1—H3···Cl1^ii^	0.86	2.34	3.1903 (15)	170
N2—H4···Cl1	0.86	2.26	3.1026 (12)	166
C2—H2···Cl1^iii^	0.97	2.78	3.7137 (14)	163
C2—H5···O1^iv^	0.97	2.57	3.5190 (18)	165
C1—O1···*Cg*1^v^	1.20 (1)	3.13 (1)	3.9430 (15)	125 (1)

Symmetry codes: (i) −*x*+1, −*y*+1, −*z*+2; (ii) *x*−1/2, −*y*+1/2, −*z*+2; (iii) −*x*+1, *y*−1/2, −*z*+3/2; (iv) −*x*+3/2, *y*−1/2, *z*; (v) *x*+1/2, *y*, −*z*+3/2.

## References

[bb1] Alkan, C., Tek, Y. & Kahraman, D. (2011). *Turk. J. Chem.* **35**, 769–777.

[bb2] Ananthamurthy, R. V. & Murthy, B. V. R. (1975). *Z. Kristallogr.* **8**, 356–367.

[bb3] Arslan, H., Flörke, U. & Külcü, N. (2003*b*). *J. Chem. Crystallogr.* **33**, 919–924.

[bb4] Arslan, H., Flörke, U. & Külcü, N. (2003a). *Acta Cryst.* E**59**, o641–o642.

[bb6] Bruker (2009). *APEX2*, *SAINT* and *SADABS*. Bruker AXS Inc., Madison, Wisconsin, USA.

[bb7] Diallo, W., Diop, L., Plasseraud, L. & Cattey, H. (2014). *Acta Cryst.* E**70**, o618–o619. 10.1107/S1600536814009246 PMC4011225 24860409

[bb8] Khongsuk, P., Prabpai, S. & Kongsaeree, P. (2015). *Acta Cryst.* E**71**, o608–o609. 10.1107/S205698901501378X PMC4571422 26396822

[bb10] Macrae, C. F., Bruno, I. J., Chisholm, J. A., Edgington, P. R., McCabe, P., Pidcock, E., Rodriguez-Monge, L., Taylor, R., van de Streek, J. & Wood, P. A. (2008). *J. Appl. Cryst.* **41**, 466–470.

[bb11] Plater, M. J. & Harrison, W. T. A. (2016). *Acta Cryst.* E**72**, 604–607. 10.1107/S2056989016005107 PMC4908533 27307999

[bb12] Rajasekaran, R., Kumar, R. M., Jayavel, R. & Ramasamy, P. (2003). *J. Cryst. Growth*, **252**, 317–327.

[bb13] Rizos, C. V., Kei, A. & Elisaf, M. S. (2016). *Arch. Toxicol.* **90**, 1861–1881. 10.1007/s00204-016-1737-4 27165418

[bb14] Saeed, A. & Flörke, U. (2006). *Acta Cryst.* E**62**, o2403–o2405.

[bb15] Saeed, A., Flörke, U. & Erben, M. F. (2014). *J. Sulfur Chem.* **35**, 318–355.

[bb16] Saeed, S., Rashid, N., Jones, P. G., Ali, M. & Hussain, R. (2010). *Eur. J. Med. Chem.* **45**, 1323–1331. 10.1016/j.ejmech.2009.12.016 20056520

[bb18] Sheldrick, G. M. (2008). *Acta Cryst.* A**64**, 112–122. 10.1107/S0108767307043930 18156677

[bb19] Singh, S. P., Parmar, S. S., Raman, K. & Stenberg, V. I. (1981). *Chem. Rev.* **81**, 175–203.

[bb20] Spek, A. L. (2009). *Acta Cryst.* D**65**, 148–155. 10.1107/S090744490804362X PMC2631630 19171970

[bb21] Ushasree, P. M., Muralidharan, R., Jayavel, R. & Ramasamy, P. J. (2000). *J. Cryst. Growth*, **218**, 365–371.

[bb22] Vedavathi, B. M. & Vijayan, K. (1981). *Acta Cryst.* B**37**, 475–477.

[bb23] Xuan, R.-C., Hu, W.-X., Yang, Z.-Y. & Xuan, R.-R. (2003). *Acta Cryst.* E**59**, o1707–o1709.

[bb24] Yamuna, T. S., Jasinski, J. P., Kaur, M., Anderson, B. J. & Yathirajan, H. S. (2014). *Acta Cryst.* E**70**, 203–206. 10.1107/S1600536814020169 PMC4257175 25484652

